# Brief Communication: Factors associated with willingness to use long-acting injectable Cabotegravir for HIV pre-exposure prophylaxis (PrEP) among female undergraduate students at a Ugandan university

**DOI:** 10.1186/s12981-024-00686-5

**Published:** 2024-12-20

**Authors:** Bridget Atuhaire, Laban Muteebwa, Racheal Nabunya, Richard Muhindo, Tom Denis Ngabirano, Charles Peter Osingada, Patience A. Muwanguzi

**Affiliations:** 1 Department of Nursing, School of Health Sciences, College of Health Sciences, Makerere University, P. O. Box 7072, Kampala, Uganda; 2 School of Medicine, College of Health Sciences, Makerere University, P. O. Box 7072, Kampala, Uganda; 3 African Center for Health Equity Research and Innovation, P. O. Box 5259, Kampala, Uganda; 4 School of Nursing, University of Minnesota School of Nursing, Weaver Densford Hall, 308 Harvard St SE, Minneapolis, MN USA

**Keywords:** Long-acting cabotegravir, Pre-exposure prophylaxis, Students, Willingness

## Abstract

**Background:**

We assessed the willingness of female students at a Ugandan public university to use long-acting Cabotegravir (CAB-LA) for HIV prevention, given their high prevalence of HIV risk behaviours.

**Methods:**

Using an online questionnaire, this cross-sectional study surveyed 346 female undergraduate students aged 18–25. Factors influencing their willingness were analysed with modified Poisson regression and robust standard errors.

**Results:**

More than half, 56.7% (95% CI: 51.4 to 61.8), were willing to use CAB-LA. Willingness was significantly associated with being sexually active in the past 3 months, using alcohol in the past 6 months, or being in the 4th year of study compared to the 1st year.

**Conclusion:**

Educational initiatives on innovative HIV prevention strategies, such as CAB-LA, should be introduced early in university students’ studies to increase awareness and acceptance.

## Background

Pre-exposure prophylaxis (PrEP) involves HIV-negative individuals using antiretroviral (ARV) drugs to prevent HIV infection before exposure. Despite its effectiveness, issues like adherence hinder its uptake [1]. In Uganda, oral PrEP is available at over 260 facilities, but its use is suboptimal among some priority groups [2]. Long-acting injectable Cabotegravir (CAB-LA) could address limitations of daily pills and has shown greater efficacy in preventing HIV compared to oral PrEP [3]. CAB-LA, administered every eight weeks, has demonstrated significant reductions in HIV infection rates in various studies [4]. However, challenges such as awareness, access, injection pain, and potential side effects persist [5]. Although CAB-LA was included in Uganda’s PrEP guidelines in 2022, its implementation has been slow [6].

Despite CAB-LA demonstrating effectiveness in clinical trials, there is limited research associated with the willingness of adolescent girls and young women in education institutional settings, such as schools and universities, to use long-acting PrEP for HIV prevention, yet this particular population is vulnerable to HIV acquisition, with a prevalence rate of 1.5% compared to 0.9% among men%) [7]. This is made worse by the predominant focus on other groups, such as men who have sex with men, cisgender individuals, and people who use drugs, ignoring this vulnerable population [8, 9]. This study specifically targets female university students because prior research has indicated a heightened risk of STIs within this demographic [10]. Awareness of injectable PrEP among high-risk adolescent girls and young women in Kampala, Uganda, is notably low, with only 3.9% reporting familiarity with the method [11]. Therefore, this low awareness not only limits their access to effective HIV prevention strategies but also perpetuates a cycle of vulnerability to HIV infection. Increasing awareness and education about PrEP is essential to empower this demographic and reduce their risk. However. this study aimed to assess the willingness of female students at a Ugandan public university to use long-acting Cabotegravir (CAB-LA) for HIV prevention.

## Methods

### Study setting and design

A cross-sectional study collected the data between April and June 2024. In total, we surveyed 346 female undergraduate students. This was determined using Cochran’s formula at an 80% power, 95% confidence level, and 0.05 precision; $\:n=\frac{{z}^{2}P(1-P)}{{e}^{2}}$ [12] The study was conducted at Makerere University College of Health Sciences in Kampala, Uganda, one of Africa’s oldest universities. Some students from urban and affluent families have access to better educational resources, while others come from rural and lower-income backgrounds. The students can access free HIV prevention services from the University hospital. We focused exclusively on female undergraduate students from the Faculty of Health Sciences at Makerere University because we believe that these students possess a greater understanding of HIV prevention strategies related to their medical training. They are expected to knowledge of CAB-LA as a PrEP strategy.

Only female undergraduate students aged 18–25 who self-reported to be HIV negative consented to participate in the study. After assessing their eligibility criteria, an online data collection tool developed using the Kobo toolbox was shared via the students’ email addresses or WhatsApp according to their preference.

We excluded those with a dead year and those absent during the study. Consecutive sampling was used to enroll participants from all the schools at Makerere University College of Health Sciences, i.e., School of Biomedical Sciences, School of Dentistry, School of Public Health, School of Health Sciences, and School of Medicine. Participants from all academic years, including years one to five, were considered for the study. The study team developed a data collection tool based on existing literature. The newly developed tool assessed willingness to use CAB-LA with three 5-level Likert scale questions based on existing literature. Scores ranged from 1 (very unwilling) to 5 (very willing), with higher scores indicating greater willingness. Scores were summed and dichotomised at the 50th percentile (score of 9), with scores above 9 indicating willingness to use CAB-LA [13].

### Data management and analysis

The data collection tool was designed using Kobo Collect software, and data analysis was done using STATA version 17.0. A modified Poisson regression analysis assessed the association between the willingness to use CAB-LA and independent variables. This method provides accurate relative risks for common events (prevalence > 10%), unlike logistic regression, which can overestimate them [14]. Unadjusted analysis estimated crude prevalence ratios (cPR) and 95% confidence intervals (CI). Independent variables with a P value < 0.2 were considered for adjusted analysis. The final model used a significance level of P value 0.05, reporting adjusted prevalence ratios (aPR) and their 95% CI.

## Results

### Demographic, sexual and behavioural characteristics of the study participants

Table 1 shows the demographic, sexual and behavioural characteristics of the female students.

**Table 1:** Demographic, sexual and behavioural characteristics of female undergraduate students at a Ugandan public university (*N* = 346)

Demographic characteristic	Categories	Frequency (*N* = 346)	(%)
Age (completed years)	Median (IQR)	23 (22, 25)	
Year of Study	1st Year	70	20.2
	2nd Year	62	17.9
	3rd Year	91	26.3
	4th Year	94	27.2
	5th Year	29	8.4
Residence status	On-Campus Resident	186	53.8
	Non-Resident	160	46.2
Religion	Christian	312	90.2
	Non-Christian	34	9.8
Sponsor	Government-sponsored	206	59.7
	Private with Scholarship	41	11.9
	Private without Scholarship	98	28.4
Marital status	Married/Live with partner	31	9.0
	Not Married	315	91.0
**Sexual and behavioural characteristics**	**Categories**	**Frequency** (***N*** = **246**)	**(%)**
Ever been pregnant	Yes	21	6.1
Ever used a contraceptive method	Yes	89	25.7
Contraceptive method previously used*	Condoms	31	34.9
	Emergency pills	42	47.2
	Injectable	1	1.1
	Intra-uterine device	5	5.6
	Natural methods	5	5.6
	Oral pills	5	5.6
Sexual activity (past 3 months^$^)	Yes	197	57.3
Number of sexual partners in the past 3 months^@^	One partner	146	74.1
	> one partner	51	25.9
Primary partner having other sexual partners^@^	Yes	19	9.7
Knowledge about HIV status of the primary partner^@^	Yes	164	83.3
HIV status of the partner^#^	Positive	1	0.6
Used injectable recreational drugs (past 6 months)	Yes	13	3.8
Ever tested for HIV (past 12 months)	Yes	227	65.6
Disclosure of HIV status to partner (s)^##^	Yes	183	81.7
Age difference with the primary partner^	< 10 years	80	41.0
Ever heard about HIV prevention methods	Yes	285	82.4
Access to preferred HIV prevention methods at place of residence	Yes	222	64.2
Alcohol use in the past 6 months	Yes	103	29.8
Perceived HIV risk	High risk	35	10.1
	Low risk	187	54.1
	Not at risk	124	35.8
Ever heard about HIV PrEP^%^	Yes	262	76.2
Ever used HIV PrEP**	Yes	9	3.4

*N = 89, ^$^*N* = 344, ^@^*N* = 197, ^#^*N* = 162, ^##^*N* = 224, ^*N* = 195, ^%^*N* = 344, ^**^N = 262, %=percentage

### Willingness to use CAB-LA and associated factors

The median willingness to use CAB-LA score was 10 (IQR: 6 to 15). Of the female university students, 56.7% (95% CI: 51.4 to 61.8) were willing to use CAB-LA.

Table 2 shows the analysis of the factors associated with willingness to use CAB-LA.

**Table 2:** Adjusted and unadjusted analysis of demographic factors associated with willingness to use CAB-LA among female students at a Ugandan public university (*N* = 346)

Variable	Categories	cPR (95% CI)	*P* value	aPR (95% CI)	*P* value
Year of Study	1st Year	Ref		Ref	
	2nd Year	1.00 (0.71, 1.41)	1.000	1.04 (0.74, 1.46)	0.825
	3rd Year	0.97 (0.71, 1.33)	0.836	0.98 (0.72, 1.34)	0.906
	4th Year	1.38 (1.06, 1.81)	**0.019**	1.34 (1.03, 1.75)	**0.032**
	5th Year	1.45 (1.05, 2.01)	**0.026**	1.31 (0.95, 1.80)	0.105
Religion	Christian	Ref			
	Non-Christian	0.65 (0.42, 1.01)	**0.055**		
Marital status	Married	Ref			
	Not married	0.92 (0.68, 1.23)	0.566		
Sexual activity - past 3 months	Not Sexually active	Ref		Ref	
	Sexually active	1.34 (1.10, 1.64)	**0.004**	1.25 (1.01, 1.53)	**0.038**
HIV test in the past 12 months	Yes	1.49 (1.18, 1.88)	**0.001**		
Knows partner’s HIV status	Yes	1.29 (0.91, 1.83)	**0.153**		
Alcohol use in the past 6 months	Yes	1.37 (1.15, 1.64)	**< 0.001**	1.24 (1.03, 1.49)	**0.023**
Self-perceived HIV risk	No HIV risk	Ref			
	Low HIV risk	1.24 (0.90, 1.72)	**0.196**		
	High HIV risk	1.27 (1.03, 1.58)	**0.029**		
Access to preferred HIV prevention methods at place of residence	Yes	0.85 (0.70, 1.02)	**0.072**		
Ever heard about HIV PrEP	Yes	1.22 (0.96, 1.56)	**0.109**		

## Discussion

This study assessed the willingness of female university students to use CAB-LA and the associated factors. Over half of the participants were willing to use CAB-LA, which is sub-optimal for large-scale implementation. Higher willingness was previously reported in studies among key and minority populations. For example, acceptability varied by demographics, with a higher preference among males, especially men who have sex with men (MSM) in the US, and females outside the US [15], as well as in Africa [16]. The differences in willingness across populations may be due to the higher prevalence and acceptance of PrEP among high-risk groups [17, 18]. Female health profession students may not perceive themselves as high risk, as only 10.1% felt they were at high risk of HIV. This highlights the need for targeted education and awareness campaigns to increase PrEP uptake among broader populations, including university students.

This study showed a higher willingness to use CAB-LA compared to a study in the US, where only 32.4% [19] were willing, as well as in Nigeria, where 50.1% of the youth (14–24 years) were willing [20]. The higher willingness in our study may be due to the participants being female and more likely to seek reproductive health services [21]. This suggests that educational initiatives about HIV prevention are effective and that there may be a supportive environment for implementing CAB-LA programs in Uganda. Future research should assess the willingness of male university students to use CAB-LA.

Fourth-year female students are 1.34 times more likely to use CAB-LA for HIV prevention compared to first-year female students. Their greater education and clinical experience enhance their understanding of HIV prevention and the benefits of long-acting PrEP. However, a study found that healthcare professional students were less willing to prescribe PrEP as they advanced in their programs [22]. Addressing this early can help develop interventions to ensure healthcare providers support PrEP use. Therefore, medical schools and policymakers should consider focusing educational interventions on HIV prevention during the early stages of training.

Sexually active female health professional students were 25% more willing to use CAB-LA compared to their non-sexually active peers. This aligns with findings from a study on oral PrEP predictors [23]. Factors contributing to this include their medical training, access to current HIV prevention information, and the convenience of less frequent dosing with Long-acting PrEP. The higher willingness among sexually active individuals may also relate to their increased HIV risk [24]. Healthcare providers should regularly discuss sexual health with university students to tailor prevention strategies. Further research should explore the broader implications of sexual practices and HIV risk, with longitudinal studies examining evolving attitudes towards Long-acting PrEP and its impact on HIV prevention.

High alcohol consumption is linked to risky sexual behaviour, with drinkers being more likely to engage in transactional sex and have multiple or concurrent partnerships [25]. The cognitive effects of alcohol impair judgment, leading to risky behaviours that increase HIV transmission risk [26]. Social factors, such as peer influence and discussions about sexual health within social circles, also play a role. Peer-led initiatives targeting those who consume alcohol could be beneficial in increasing awareness and willingness to use HIV prevention measures like CAB-LA [27].

The study’s focus on health profession students at one public university limits its generalizability. Future research should use longitudinal designs.

## Data Availability

The datasets used or analysed during the current study are available from the corresponding author upon reasonable request.
